# Comparative Effects of Heterologous TRPV1 and TRPM8 Expression in Rat Hippocampal Neurons

**DOI:** 10.1371/journal.pone.0008166

**Published:** 2009-12-04

**Authors:** Devon C. Crawford, Krista L. Moulder, Robert W. Gereau, Gina M. Story, Steven Mennerick

**Affiliations:** 1 Graduate Program in Neurosciences, Washington University, St. Louis, Missouri, United States of America; 2 Department of Psychiatry, Washington University, St. Louis, Missouri, United States of America; 3 Department of Anesthesiology, Washington University, St. Louis, Missouri, United States of America; 4 Department of Anatomy and Neurobiology, Washington University, St. Louis, Missouri, United States of America; SIU School of Medicine, United States of America

## Abstract

Heterologous channel expression can be used to control activity in select neuronal populations, thus expanding the tools available to modern neuroscience. However, the secondary effects of exogenous channel expression are often left unexplored. We expressed two transient receptor potential (TRP) channel family members, TRPV1 and TRPM8, in cultured hippocampal neurons. We compared functional expression levels and secondary effects of channel expression and activation on neuronal survival and signaling. We found that activation of both channels with appropriate agonist caused large depolarizing currents in voltage-clamped hippocampal neurons, exceeding the amplitude responses to a calibrating 30 mM KCl stimulation. Both TRPV1 and TRPM8 currents were reduced but not eliminated by 4 hr incubation in saturating agonist concentration. In the case of TRPV1, but not TRPM8, prolonged agonist exposure caused strong calcium-dependent toxicity. In addition, TRPV1 expression depressed synaptic transmission dramatically without overt signs of toxicity, possibly due to low-level TRPV1 activation in the absence of exogenous agonist application. Despite evidence of expression at presynaptic sites, in addition to somatodendritic sites, TRPM8 expression alone exhibited no effects on synaptic transmission. Therefore, by a number of criteria, TRPM8 proved the superior choice for control over neuronal membrane potential. This study also highlights the need to explore potential secondary effects of long-term expression and activation of heterologously introduced channels.

## Introduction

There has been strong recent interest in heterologous control over electrical activity in neurons and other excitable cells [1], [2], [3]. These approaches offer the possibility of remotely controlling the activity of select populations of neurons, thereby gaining experimental or therapeutic influence over network activity. For instance, heterologously introduced channels have been used recently to control courtship and escape behavior in flies and fish [4], [5], [6], [7] and to control sleep-wake behavior and motor behavior, including Parkinsonian and epileptic symptoms, in mammals [8], [9], [10], [11], [12], [13]. Because electrical activity is critical for neuronal survival, development, and plasticity [14], [15], [16], these techniques also have experimental potential to help unravel the downstream signaling mechanisms responsible for these important facets of neuronal function [17], [18].

Heterologous expression of ligand-gated receptors and leak channels received initial attention [17], [19], [20], [21], [22], but this approach has largely been supplanted by optogenetic approaches that employ channels directly activated by light [23], [24], [25], [26]. Multiple approaches for heterologous control over activity are probably needed, however, as one single method is unlikely to be appropriate in all situations. Introduction of leak channels [17], [21], [22] suffers from a lack of control over the degree of activity change and typically is limited to an inhibitory influence, and one obvious disadvantage of optogenetic approaches is the need for a light source. A light source of appropriate wavelength will not be feasible or cost-effective in all circumstances. In the intact nervous system, light implantation may be prohibitively invasive. Furthermore, some applications may require activation of a spatially dispersed set of neurons over a large area, in which case a single light source may not be adequate.

For these reasons, we have explored further the advantages and disadvantages of a heterologous ligand-gated receptor approach. This approach offers the possibility of global administration of ligand for activating populations of heterologously transfected neurons. We explored two candidate heterologous ligand-gated channels: transient receptor potential vanilloid receptor 1 (TRPV1) and transient receptor potential cation channel, subfamily M, member 8 (TRPM8), which normally are responsible for pain and temperature signal transduction in sensory neurons and respond to the pharmacological ligands capsaicin and menthol, respectively [27], [28], [29]. These channels were chosen for their ability to be controlled readily by agonist application and their lack of strong endogenous role in hippocampal function. To test advantages and disadvantages of expressing each channel in hippocampal neurons, we used sparse transfection of neurons to avoid complications and confounds of widespread neuronal activation and associated release of neurotransmitters and modulators. We tested the activation of these channels relative to depolarizing currents activated by elevated extracellular potassium as a calibration standard.

Acute activation of both TRPV1 and TRPM8 induced large currents in response to saturating agonist concentration. Both showed evidence of desensitization with prolonged receptor activation, although these responses still compared favorably with non-desensitizing potassium-induced depolarizing currents. TRPM8 exhibited less agonist-induced toxicity and less interference with normal synaptic communication in the absence of agonist. In addition to soma expression of channels, we observed evidence for direct depolarization of axon terminals of TRPM8- and TRPV1-transfected neurons, suggesting cell-wide expression. Because our results suggest that TRPM8 is expressed throughout the cell with no detectable secondary effects on cell function, TRPM8 is the better choice for studies requiring exogenous control over the membrane potential of neuronal subpopulations. Our results also stress the importance of fully characterizing a heterologous expression system to avoid confounding and other unintended effects of manipulating that system.

## Results

### TRPV1 transfection

**Figure 1:**
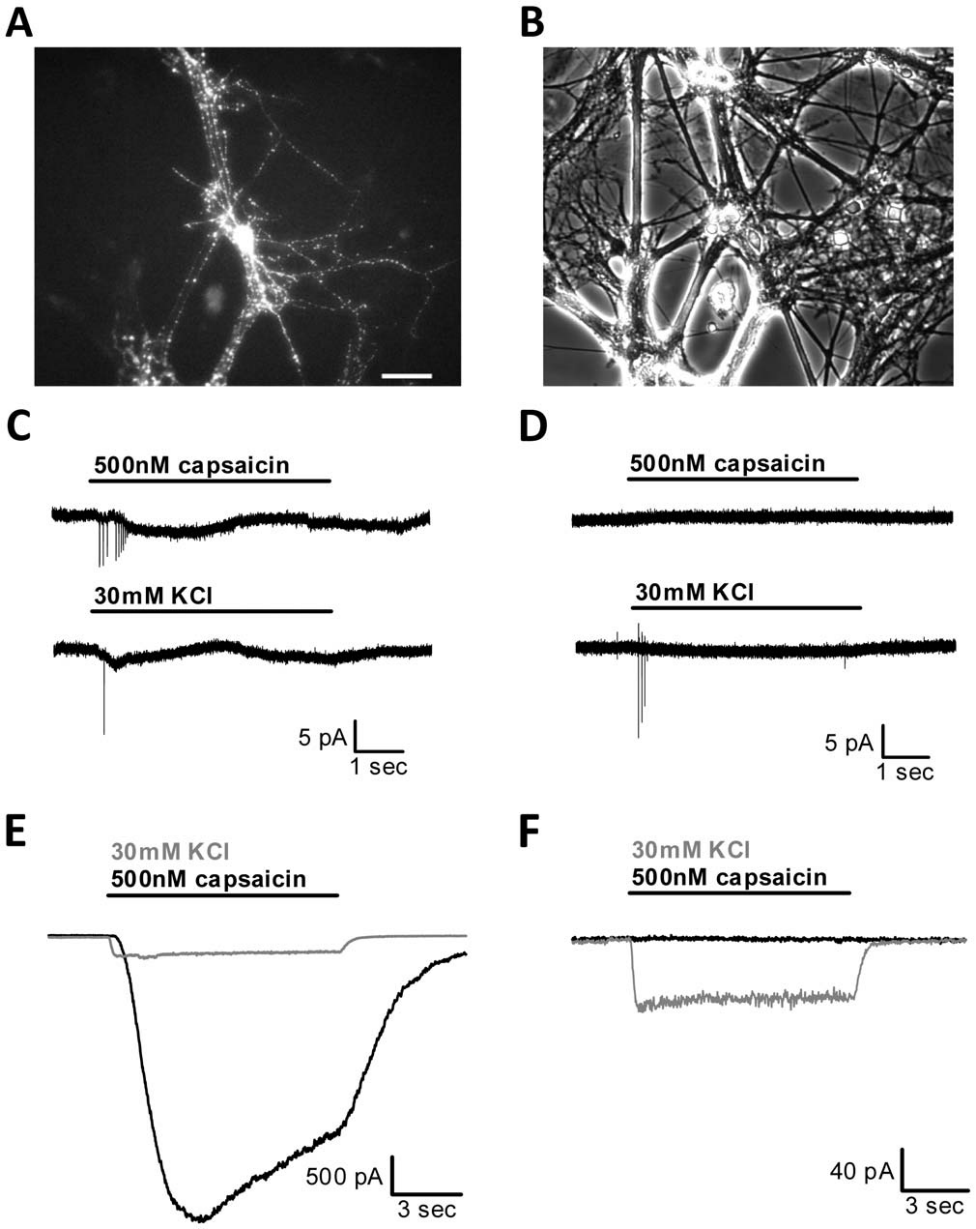
TRPV1-transfected neurons respond strongly to the agonist capsaicin. (A) Fluorescence image of a neuron in mass culture transfected with synaptophysin-YFP (Syn-YFP) and TRPV1. Scale bar denotes 100 µm. (B) Phase contrast image of the same field as (A). (C) Example of a Syn-YFP/TRPV1-transfected neuron recorded in cell-attached patch configuration in voltage-clamp mode during 5 s 500 nM capsaicin application (top trace) or 5 s 30 mM KCl application (bottom trace). Note that action potentials could be elicited by both treatments in the same cell. The suboptimal seal, evident in the slow current excursions, was characteristic of nearly all TRPV1-transfected neurons. (D) Example of a non-transfected neuron recorded under the same conditions as (C). Note that action potentials were only elicited during KCl application. (E) Example of a Syn-YFP/TRPV1-transfected neuron recorded in whole-cell voltage-clamp mode during 10 s acute 500 nM capsaicin or 30 mM KCl application. TRPV1-transfected neurons responded with robust currents to both capsaicin and KCl application. (F) Example of a non-transfected neuron recorded under the same conditions as (E). Non-transfected neurons never responded to capsaicin but always responded with robust KCl-induced currents.

We transfected neurons in our primary mass hippocampal cultures with heterologous ligand-gated ion channels to spatially and temporally control neuronal depolarization. We co-transfected a yellow fluorescent protein-tagged synaptophysin construct (Syn-YFP), which served as a marker for somata and presynaptic terminals in transfected neurons (Figure 1A). We first co-expressed the ligand-gated, nonselective cation channel TRPV1 (Figure 1A and 1B). Whole-cell currents (see Figure 1E and 1F) from YFP-positive and YFP-negative neurons in cultures co-transfected with TRPV1 showed that, at a 1∶4 Syn-YFP∶TRPV1 DNA ratio, no neurons expressed one construct without the other (N = 38 transfected and 19 non-transfected neurons). Assessed by YFP fluorescence, transfection efficiency was 1.1±0.3% (N = 3 dishes).

To determine whether current amplitudes generated by TRPV1 were sufficiently large to cause action potentials in transfected cells, we performed cell-attached patch recordings from neurons. Cell-attached patch methods were used in order to prevent cell rupture, which would cause cytosolic mixing with internal patch pipette solution and, therefore, potentially alter the intracellular milieu and neuronal responses to environmental manipulation. Application of a saturating concentration (500 nM) of the TRPV1 agonist capsaicin [27], [30], which was chosen in order to maximize neuronal depolarization and potential secondary effects of channel activation, induced a pattern of 1–9 action potentials in TRPV1-expressing neurons but no action potentials in non-transfected neurons (Figure 1C and 1D; N = 15 transfected and 5 non-transfected neurons). From the same cells, 30 mM KCl application caused 1–6 action potentials in both transfected and non-transfected neurons (Figure 1C and 1D; N = 19 neurons). Hippocampal neurons are capable of sustaining action potential firing for periods of at least as long as our 5 s agonist application shown in Figure 1C, but spiking typically ceased soon after the start of capsaicin application. This may result from small or rapidly desensitizing capsaicin-induced currents or from very large currents that cause strong inactivation of the sodium channels necessary for spiking. This latter explanation almost certainly applied to the effects of KCl, a strong depolarizing stimulus [31], [32], [33].

To determine if rapid desensitization of capsaicin-induced current is responsible for abbreviated spiking in response to capsaicin, we examined capsaicin- and KCl-elicited currents in voltage-clamped neurons. YFP-positive neurons, unlike non-transfected neurons (5.3±4.4 pA; N = 4), reliably responded to the TRPV1 agonist capsaicin with robust (−1385.7±574.8 pA; N = 6) currents measured via whole-cell voltage clamp (Figure 1E and 1F). As a comparison, we examined the response to current induced by 30 mM KCl application, which is known to clamp the membrane potential at steady state near −20 mV [32]. Non-transfected neurons responded with a KCl-induced current amplitude of -159.5±62.3 pA (N = 4) while transfected neurons responded with a similar amplitude of -204.0±30.8 pA (N = 6). The capsaicin current desensitized but remained large during the application period, suggesting that rapid desensitization of capsaicin-induced current is not likely to be responsible for the abbreviated spiking observed in cell-attached patch experiments. The robust sustained current in capsaicin, however, does implicate voltage-gated sodium channel inactivation or other voltage-dependent processes as a possible explanation. We conclude that currents elicited by capsaicin in transfected neurons are similar to or larger on average than those elicited by KCl and, therefore, that 500 nM capsaicin elicits a strong yet selective depolarization in TRPV1-transfected neurons.

These observations were confirmed by neuronal responses to exogenous compounds recorded in whole-cell current-clamp, where transfected neurons produced a burst of 4–6 action potentials in response to 5 s 500 nM capsaicin and depolarized to a steady state of −4.0±1.7 mV (N = 5) while non-transfected neurons did not respond to capsaicin (N = 5; Figure S1A, S1B; Text S1). Both transfected and non-transfected neurons produced a burst of 0 (in 1/10 cells) to 9 action potentials in response to 5 s 30 mM KCl while the membrane potential depolarized to a non-steady state value of −33.9±3.4 mV during application. These results support the conclusion that acute application of capsaicin to transfected neurons can meet or exceed the strength of depolarization achieved by a calibrating KCl stimulation.

**Figure 2:**
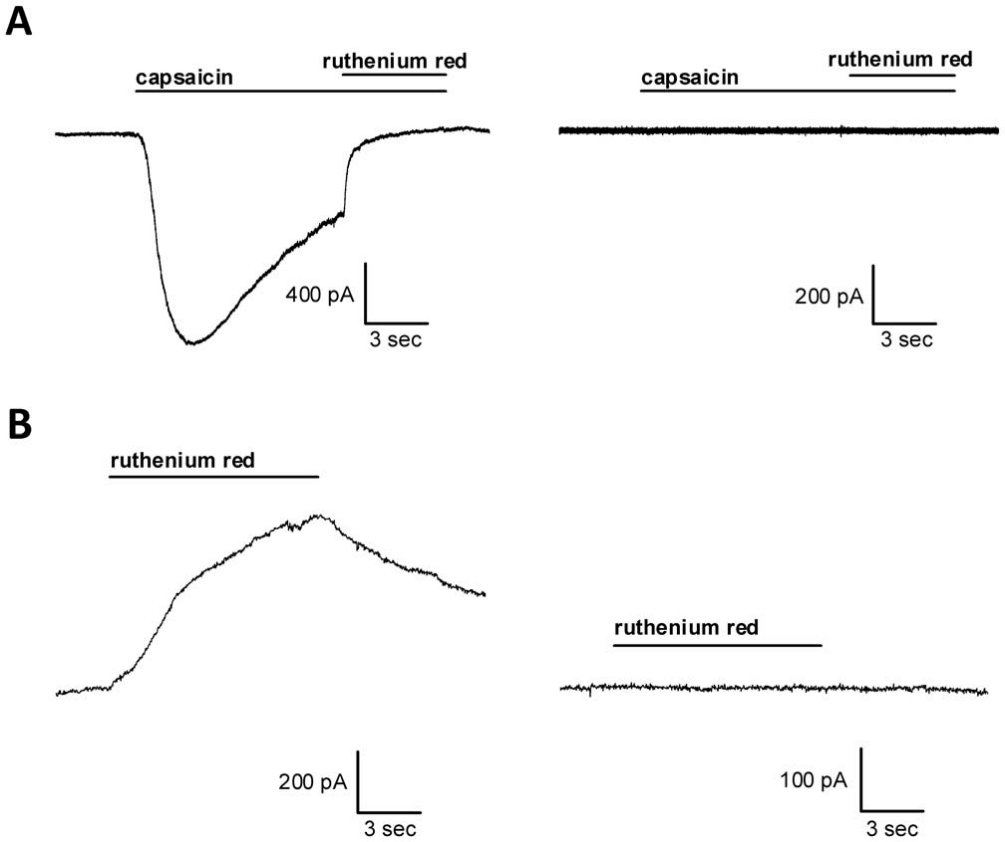
Capsaicin-induced and capsaicin-independent currents are blocked by ruthenium red in TRPV1-transfected neurons. (A) Examples of TRPV1-transfected (left panel) and non-transfected (right panel) neurons exposed to acute application of 500 nM capsaicin for 15 s with 10 µM ruthenium red added during the last 5 s. TRPV1-transfected neurons, but not non-transfected neurons, respond to capsaicin with a robust current that is blocked by ruthenium red. (B) Examples of TRPV1-transfected (left panel) and non-transfected (right panel) neurons exposed to acute application of 10 µM ruthenium red in the absence of capsaicin. Leaky (more than −200 pA standing inward current) TRPV1-transfected neurons reliably responded to ruthenium red while non-leaky TRPV1-transfected neurons and non-transfected neurons did not.

We noticed that TRPV1-transfected neurons tended to have larger basal holding currents (−177.7±60.9 pA; N = 30) than control neurons (−51.1±10.0 pA; N = 32; Student's unpaired *t* test, p<0.05) when voltage-clamped at −70 mV. We wondered whether this observation could reflect non-ligand-gated channel opening of TRPV1. We tested this explicitly by applying the non-competitive TRPV1 antagonist ruthenium red (10 µM), which blocked 91.8±5.3% of the capsaicin-gated current in our TRPV1-transfected (500 nM capsaicin; N = 11) but caused no effect in non-transfected (N = 8) neurons (Figure 2A). When we applied 10 µM ruthenium red to agonist-naïve, TRPV1-transfected neurons at the holding potential of −70 mV, we noticed that ruthenium red blocked standing inward current, especially in the leakiest (>−200 pA) TRPV1-transfected cells (Figure 2B). Ruthenium red blocked 12.3±3.1% (N = 15) of standing inward current in TRPV1-transfected neurons while not affecting non-transfected neurons (1.5±1.8%; N = 9; Student's unpaired *t* test, p<0.05). This suggests that at least some TRPV1-transfected neurons exhibit capsaicin-independent channel openings, even in defined recording media, which could be detrimental to the goal of controlling TRPV1-mediated depolarization.

### TRPM8 transfection

**Figure 3:**
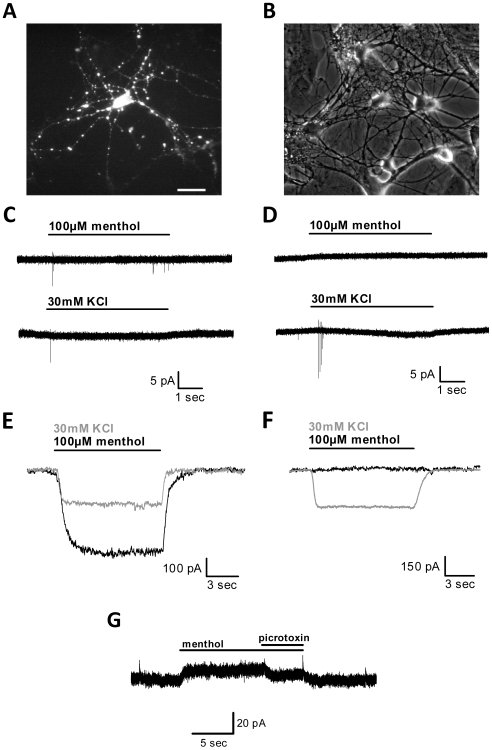
Menthol induces large depolarizing currents in TRPM8-transfected neurons. (A) Fluorescence image of a neuron in mass culture transfected with synaptophysin-YFP (Syn-YFP) and TRPM8. Scale bar denotes 50 µm. (B) Phase contrast image of the same field as (A). (C) Example of a Syn-YFP/TRPM8-transfected neuron recorded in cell-attached patch configuration in voltage-clamp mode during 5 s 100 µM menthol application (top trace) or 5 s 30 mM KCl application (bottom trace). Note that action potentials were elicited by both treatments in the same cell. (D) Example of a non-transfected neuron recorded under the same conditions as (C). Note that action potentials were only elicited during KCl application. (E) Example of a Syn-YFP/TRPM8-transfected neuron recorded in whole-cell voltage-clamp during 10 s acute 100 µM menthol or 30 mM KCl application. TRPM8-transfected neurons responded with robust currents to both menthol and KCl application. (F) Example of a non-transfected neuron recorded under the same conditions as (E). Non-transfected neurons did not respond to menthol but always responded with robust currents to KCl at the holding potential of −70 mV. (G) A non-transfected neuron recorded in whole-cell voltage clamp held at 0 mV during 15 s acute application of 100 µM menthol with 100 µM picrotoxin added during the last 5 s. Note that picrotoxin decreased the current gated by menthol.

We also transfected cultured hippocampal neurons with the ligand-gated nonselective cation channel TRPM8 (Figure 3A and 3B). At a 1∶3 Syn-YFP∶TRPM8 DNA ratio, we confirmed that transfected neurons responded to acute application of the TRPM8 agonist menthol at a saturating concentration (100 µM; N = 38) [28], [34], once again chosen to maximize effects of channel activation, while non-transfected cells did not respond (N = 10). Similar to the experiments above with TRPV1, transfection efficiency was 1.2±0.4% as assessed by YFP fluorescence (N = 3 dishes).

YFP-positive neurons recorded in the cell-attached patch configuration responded with a pattern of 1–7 action potentials during 100 µM menthol application, as they did during KCl application (Figure 3C; N = 13 transfected neurons). These action potential responses were similar to those seen in TRPV1-transfected neurons (Figure 1C), and non-transfected neurons responded to KCl with 1–8 action potentials but did not respond to menthol (Figure 3D; N = 26 non-transfected neurons). When we applied these exogenous compounds while recording from TRPM8-transfected neurons in current-clamp, we again observed bursts of 1–6 action potentials and a steady state membrane potential of −13.1±6.2 mV elicited by 5 s 100 µM menthol application (N = 6; Figure S1C; Text S1). Non-transfected neurons did not respond to menthol application (N = 5; Figure S1D). In these experiments, 30 mM KCl consistently elicited 1–3 action potentials in all cells and a membrane potential after 5 s, though not yet at steady state, of −37.1±1.9 mV. TRPM8-transfected neurons exhibited large inward currents measured in voltage-clamp during acute menthol application (−396.4±69.6 pA; N = 28) while YFP-negative neurons did not respond with inward current (23.9±20.1 pA; N = 10) at −70 mV (Figure 3E and 3F). As seen with TRPV1-transfected neurons, these menthol-induced currents in TRPM8-transfected neurons were similar to or larger than currents induced by 30 mM KCl (−153.4±27.8 pA; N = 8 transfected neurons; −326.6±96.2 pA; N = 10 non-transfected neurons). We conclude that both TRPV1 and TRPM8 generate sufficiently large currents in transfected hippocampal neurons to cause strong, sustained (for several seconds) depolarization with acute agonist application, equivalent to or larger than the effects of 30 mM KCl application.

Standing inward current in TRPM8-transfected neurons (−44.7±16.9 pA; N = 32) was not significantly different from non-transfected cells (−57.2±36.5 pA; N = 8), suggesting that TRPM8 does not suffer from the same non-ligand-gated channel opening as TRPV1. However, in some non-transfected cells held at 0 mV, we observed a small sustained outward current in the presence of 100 µM menthol (13.1±1.3 pA; Figure 3G; N = 8). This current was observed even in the presence of 0.5 µM TTX and changed direction appropriately with alterations in the chloride gradient (Figure S2; Text S1). This latter result excludes the possibility that the current is mediated by endogenous TRPM8 in non-transfected neurons, and no published evidence currently indicates endogenous TRPM8 expression in rodent hippocampus. A previous report suggests, however, that menthol can directly gate GABA_A_ receptor-mediated chloride currents in hippocampal neurons [35]. Picrotoxin (100 µM), a noncompetitive GABA_A_ receptor antagonist, reduced the outward current in non-transfected neurons by 85.4±12.9% (Figure 3G; N = 8 neurons). Therefore, these data provide evidence that menthol may weakly gate endogenous GABA_A_ receptors in cultured neurons.

### Prolonged channel activation

To control neuronal activity and evaluate the effects on development, synaptic function, or network activity, it may be desirable to depolarize neurons for long periods of time. For this reason, we activated TRPV1 and TRPM8 in cultured neurons for 4 hr to determine if depolarization was sustained throughout the period of agonist exposure. We chose to activate these channels for hours, instead of a shorter time period, to account for slow forms of channel desensitization or inactivation that may be induced (i.e. by cellular adaptation). These slower changes may be important considerations for studies using heterologous channels *in vivo* or for studies of neuronal circuit activity that utilize changes in activity of subsets of neurons for long periods. To activate TRPV1, we added 500 nM capsaicin to the culture media in the presence of D-APV (25 µM) and NBQX (1 µM) while the neurons remained at 37°C in an atmospherically-controlled incubator. Similarly, TRPM8-transfected cultures were exposed to 100 µM menthol under the same conditions.

**Figure 4:**
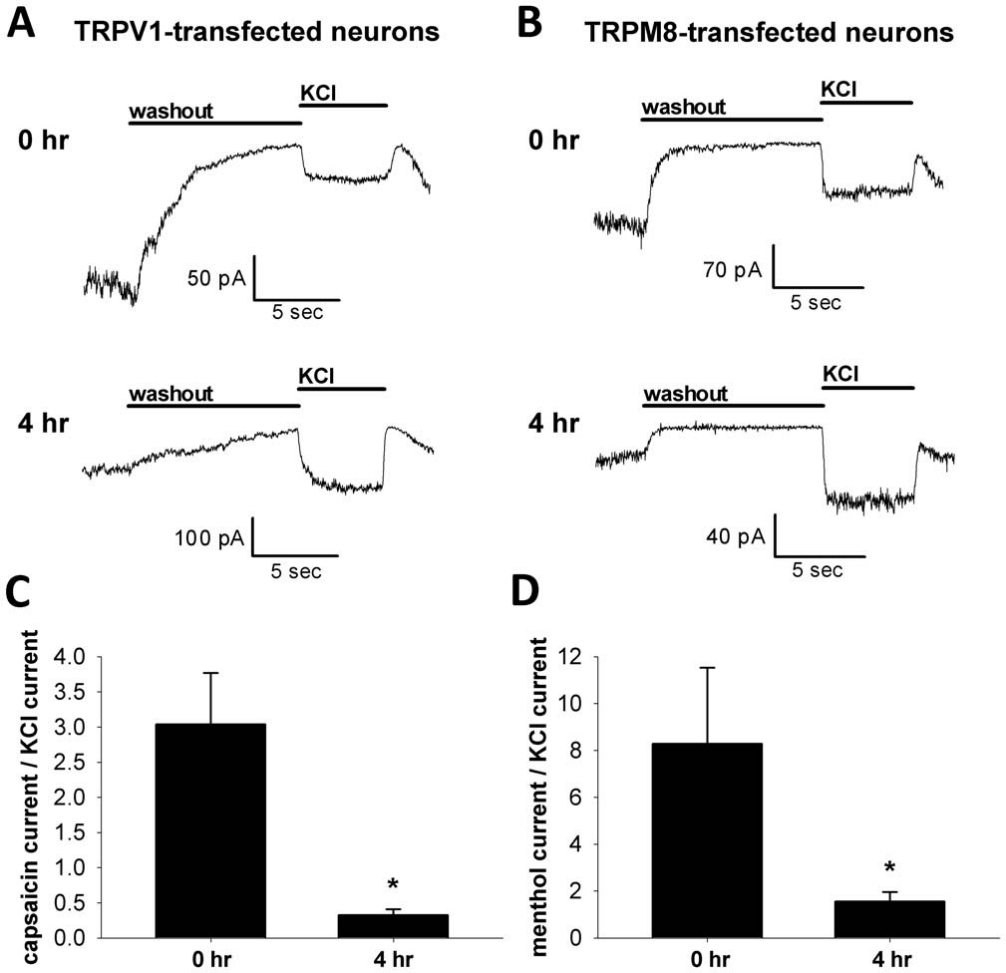
Agonist-induced currents in both TRPV1- and TRPM8-transfected neurons are decreased after prolonged exposure. (A) Examples of TRPV1-transfected neurons exposed to bath application of 500 nM capsaicin, 25 µM D-APV, and 1 µM NBQX acutely (top panel) or for 4 hr (bottom panel) prior to recording in whole-cell voltage clamp. Capsaicin was retained in the bath recording solution. After the whole-cell recording was established, capsaicin was briefly removed by perfusing fresh recording saline for 10 s before 5 s application of 30 mM KCl. (B) Examples of TRPM8-transfected neurons exposed to bath application of 100 µM menthol, 25 µM D-APV, and 1 µM NBQX acutely (top panel) or for 4 hr (bottom panel) prior to recording in whole-cell voltage clamp. Similar to (A), menthol was added to the bath recording solution. Menthol was removed by perfusing fresh, menthol-free recording saline for 10 s before 5 s application of 30 mM KCl. (C) The average ratio of capsaicin current amplitude to KCl current amplitude was calculated for acute and 4 hr applications of capsaicin. Error bars represent SEM. The ratio of capsaicin to KCl current is significantly decreased after 4 hr capsaicin exposure (Student's unpaired *t* test, p<0.05). (D) Similar to C except the ratio of menthol to KCl currents in TRPM8-transfected neurons is depicted. Error bars represent SEM, and the menthol to KCl current ratio is significantly decreased after 4 hr exposure to menthol (Student's unpaired *t* test, p<0.05).

At the end of incubation, neurons were recorded in the continued presence of agonist, and agonist-induced currents were determined by removing the agonist with a fresh saline wash and measuring the change in current amplitude. This amplitude was then compared to the current amplitude of an acute application of 30 mM KCl in the same cell (Figure 4A and 4B). Compared to brief (0–30 min) application of agonist, normalized current amplitudes for both TRPV1- and TRPM8-transfected neurons were significantly decreased after 4 hr application of their respective agonists (Figure 4C and 4D). The ratio of capsaicin current to KCl current in TRPV1-transfected neurons decreased from 3.04±0.73 (N = 32 neurons treated acutely with capsaicin) to 0.32±0.08 (N = 11 neurons treated 4 hr with capsaicin; Student's unpaired *t* test, p<0.05). The ratio of menthol current to KCl current in TRPM8-transfected neurons decreased from 8.28±3.24 (N = 7 neurons treated acutely with menthol) to 1.56±0.40 (N = 10 neurons treated 4 hr with menthol; Student's unpaired *t* test, p<0.05). The inward current present after 4 hr, however, was still comparable to the current induced by acute 30 mM KCl. In both acute and prolonged protocols, the average KCl-normalized menthol current in TRPM8-transfected neurons trended toward being larger than the corresponding normalized capsaicin current in TRPV1-transfected cells. In summary prolonged agonist application produced sustained, effective depolarization for both channels, although the amplitude was reduced with time.

**Figure 5:**
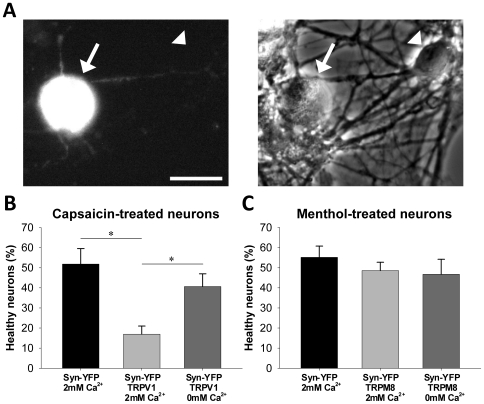
Agonist-induced toxicity in transfected neurons. (A) An example TRPV1-transfected neuron designated “unhealthy” (arrow) in the same field as a “healthy” non-transfected neuron (arrowhead). Syn-YFP fluorescence (left panel) and phase contrast (right panel) images are shown. Note the swollen cell body of the unhealthy cell. Scale bar represents 30 µm. (B) Transfected neurons treated 4 hr with 500 nM capsaicin, 25 µM D-APV, and 1 µM NBQX in culture media with (2 mM) or without (0 mM plus 500 µM EGTA) calcium were assessed as “healthy” or “unhealthy” by a naïve observer. Significantly fewer TRPV1-transfected neurons treated in calcium were healthy than either YFP-transfected neurons treated in calcium or TRPV1-transfected neurons treated without calcium (ANOVA, p<0.01; post hoc Tukey test, p<0.05). (C) Transfected neurons treated 4 hr with 100 µM menthol, 25 µM D-APV, and 1 µM NBQX in culture media with or without calcium were assessed as “healthy” or “unhealthy” as in (B). There was no significant change in health of TRPM8-transfected neurons compared to YFP-transfected neurons regardless of whether or not they were exposed to extracellular calcium. Error bars represent SEM.

The smaller average agonist-gated current after prolonged exposure probably results partly from receptor desensitization. However, it is also possible that strong, prolonged channel activation is toxic to neurons, and the smaller average currents arise as a result of selective survival of cells with weaker channel expression and smaller currents. Indeed, especially in TRPV1-transfected cultures, prolonged agonist exposure resulted in many unsuccessful electrophysiological recordings of cells with abnormally swollen morphology. To determine more systematically whether prolonged channel activation affects neuronal health, mass cultures were transfected with Syn-YFP control DNA with or without TRPV1 or TRPM8. Neurons were assigned a binary (“healthy” or “unhealthy”) survival designation by a naïve observer using cell appearance under phase contrast optics. Healthy neurons had a phase-bright soma and thin neurites. Unhealthy neurons had distorted or swollen somata and neurites (Figure 5A). In some cases, classification was verified by patch-clamp recordings; membrane seals were never possible on neurons designated unhealthy.

Neurons were assessed for health after 4 hr of channel activation using the same treatment protocol as the electrophysiology experiments in Figure 4. It is possible that transfection alone caused some baseline toxicity; however, after 4 hr of capsaicin exposure, the percentage of healthy TRPV1-transfected neurons was significantly decreased from control cultures transfected with only Syn-YFP (Figure 5B; ANOVA, p<0.01; post hoc Tukey honestly significant difference test, p<0.05; N = 5 dishes per condition with each dish representing 19.9±1.3 transfected neurons). In contrast, a 4 hr menthol challenge to TRPM8-transfected neurons caused no significant change in the percentage of healthy neurons relative to sibling cultures transfected with Syn-YFP (Figure 5C; N = 5 dishes per condition with each dish representing 22.3±2.4 transfected neurons).

Calcium influx may differ between transfected neurons due to differences in TRPV1 and TRPM8 calcium permeability [27], [28]. Since calcium influx is known to contribute to some forms of cellular toxicity [36], [37], [38], we wished to determine if toxicity in TRPV1-transfected neurons after prolonged channel activation was due to calcium influx. We assayed toxicity after 4 hr of agonist exposure in calcium-free extracellular media supplemented with 500 µM of the calcium chelator ethylene glycol-bis (β-aminoethyl ether)-*N, N, N* ′, *N* ′-tetraacetic acid (EGTA). Calcium-free media protected TRPV1-expressing neurons exposed to 500 nM capsaicin compared to those in calcium-containing media (Figure 5B); neurons exposed to capsaicin in calcium-free media appeared similar to control neurons not expressing TRPV1. Survival of TRPM8-expressing neurons exposed to 100 µM menthol in calcium-free media did not differ from either Syn-YFP-transfected controls or from TRPM8-transfected neurons treated in calcium-containing media (Figure 5C). This suggests that calcium influx during TRPV1 activation, but not during TRPM8 activation, is toxic to neurons.

### Effects of channel expression on synaptic transmission

**Figure 6:**
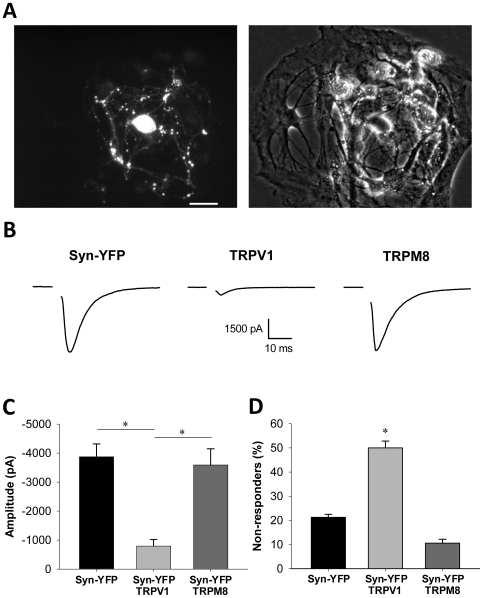
TRPV1 transfection, but not TRPM8 transfection, alters synaptic transmission. (A) Fluorescence (left panel) and phase contrast (right panel) images of a single-neuron (autaptic) island in microisland culture transfected with synaptophysin-YFP (Syn-YFP). Scale bar denotes 50 µM. (B) Representative examples of excitatory postsynaptic currents (EPSCs) evoked in transfected autaptic neurons 1 day after transfection. Stimulus artifact has been blanked in each trace for clarity. Note that TRPV1-transfected neurons produced EPSCs with smaller amplitudes than TRPM8-transfected neurons or Syn-YFP-transfected controls. (C) Quantification of average PSC amplitudes for transfected autaptic neurons. Error bars represent SEM. TRPV1-transfected neurons produced significantly smaller evoked PSCs than Syn-YFP-transfected or TRPM8-transfected neurons (ANOVA, p<0.01; Tukey-Kramer method, p<0.01). (D) Percentage of transfected autaptic neurons that did not respond to electrical stimulation with a detectable PSC. When non-responders were analyzed as “failures” from a binomial distribution, the percentage of TRPV1-transfected neurons that successfully responded to stimulation was significantly decreased from YFP-transfected controls (binomial test, p<0.01). Error bars represent SEM based on the binomial distribution.

For the utility of heterologous channels to be realized, it is important to ensure that channel introduction alone does not alter endogenous neuronal function outside of intended experimental manipulations. To determine if expression of heterologous ion channels alters synaptic properties of cultured hippocampal neurons, we transfected neurons in microisland culture with TRPV1 or TRPM8. Microisland cultures, unlike mass cultures, contain isolated “islands” of astrocytes with neurons plated on top (Figure 6A). Measuring the autaptic responses of single neurons that synapse only onto themselves is a useful method for studying synaptic efficacy in the absence of polysynaptic complications [39]. Only a minority of island cultures contains a solitary autaptic cell. Because of this low percentage, combined with low transfection efficiency, we pooled synaptic responses from glutamatergic and GABAergic cells. We used KCl internal pipette solution to create a driving force of approximately 70 mV for both excitatory postsynaptic currents (EPSCs) and inhibitory postsynaptic currents (IPSCs) at the holding potential of −70 mV.

TRPV1-transfected autaptic neurons exhibited action potential-evoked autaptic PSC amplitudes (−790.9±228.3 pA; N = 9) that were significantly decreased from Syn-YFP-transfected controls (−3870.9±444.1 pA; N = 28; Figure 6B and 6C; ANOVA, p<0.01; Tukey-Kramer method, p<0.01). TRPM8-transfected autaptic neurons, however, exhibited PSC amplitudes of −3590.8±557.3 pA (N = 17), which were comparable to Syn-YFP-transfected controls (Figure 6B and 6C). In addition to smaller amplitudes of evoked PSCs, we observed that more cells in TRPV1-transfected cultures exhibited no PSC in response to action potential stimulation. In autaptic neurons, 50% of TRPV1-transfected neurons failed to produce a response, which was significantly more than Syn-YFP-transfected controls (21%; Figure 6D; binomial test, p<0.01; N = 18 TRPV1-transfected and 33 Syn-YFP-transfected autaptic neurons recorded in microisland culture). With a failure rate of 11%, TRPM8-transfected neurons were not significantly different from controls (N = 19 TRPM8-transfected neurons). This phenomenon appears to be an extension of the observation of smaller evoked PSCs in TRPV1-transfected neurons. In summary TRPV1 transfection, in the absence of added ligand, depressed evoked synaptic transmission in autaptic hippocampal neurons whereas TRPM8 transfection had no effect.

**Figure 7:**
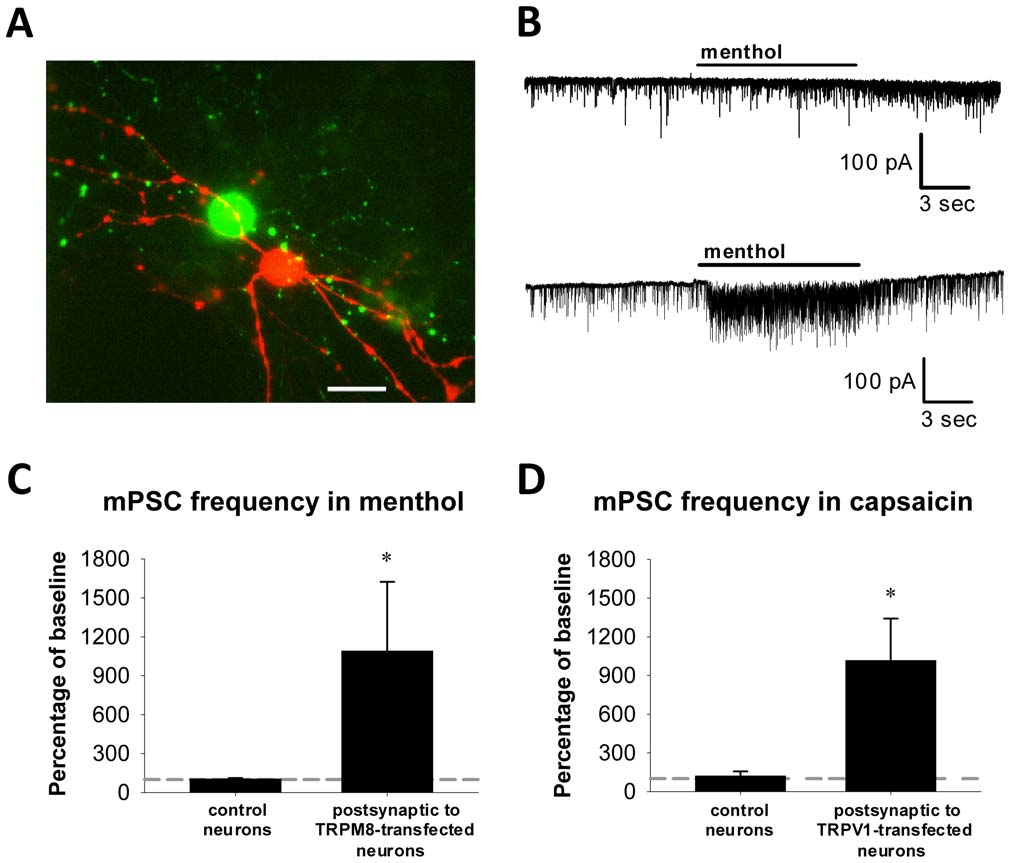
Agonist treatment in 0.5 µM tetrodotoxin (TTX) increases presynaptic transmitter release in transfected neurons. (A) Fluorescence image of a TRPM8-transfected neuron (green) forming presumed presynaptic contacts onto a postsynaptically-recorded cell (red) filled with Alexa Fluor 568, which was included in the recording pipette. Scale bar denotes 50 µM. (B) Example of miniature excitatory postsynaptic currents (mEPSCs) recorded from non-transfected postsynaptic partners of TRPM8-transfected neurons (bottom panel) or from non-transfected neurons not in contact with transfected neurons (top panel) in 0.5 µM TTX. During 100 µM menthol application, mEPSC and mIPSC frequency increased in neurons postsynaptic to TRPM8-transfected neurons but not in control neurons. (C) Quantification of the mPSC frequency during 100 µM menthol application compared to the baseline frequency (dashed line). Error bars represent SEM. In neurons postsynaptic to TRPM8-transfected neurons, but not in control neurons, the mPSC frequency increased significantly in menthol (Fisher's exact test, p<0.005). (D) Quantification of the mPSC frequency during 500 nM capsaicin application compared to baseline frequency (dashed line) in neurons postsynaptic to TRPV1-transfected neurons or to control neurons. Error bars represent SEM. In neurons postsynaptic to TRPV1-transfected neurons, but not to non-transfected neurons, the mPSC frequency in capsaicin increased significantly (Fisher's exact test, p<0.01).

One possible reason that TRPM8 does not interfere with synaptic transmission is that neurons may not express the channel at synaptic sites. TRPM8 seems targeted robustly to the somatodendritic compartment as evidenced by currents recorded from the soma (Figure 3E). Whether the channel is targeted to presynaptic terminals is less clear. If it is, axon terminal expression of TRPM8 could also be useful for probing functional connections between neurons of disparate brain regions. We used a functional test of presynaptic TRPM8 expression by investigating if TRPM8 drives synaptic neurotransmitter release independent of action potential propagation. We recorded from non-transfected target cells apposed to Syn-YFP-positive puncta (Figure 7A). We applied 100 µM menthol to neurons in the presence of 0.5 µM TTX to block action potential-driven transmitter release. If agonist-induced miniature EPSCs (mEPSCs) were not observed at a holding potential of −70 mV when using a cesium methanesulfonate internal pipette solution, then the holding potential was switched to 0 mV to record miniature IPSCs (mIPSCs). Baseline mPSC frequency, which presumably arose from mEPSCs and mIPSCs from both transfected and non-transfected presynaptic terminals, was highly variable in postsynaptic targets of both TRPM8-transfected (0.1–119.8 Hz) and control (0.4–108.0 Hz) neurons. Therefore, we expressed the changes in frequency that occurred during menthol application as a percentage of baseline frequency. Acute menthol application to TRPM8-transfected neurons (N = 26) increased mPSC frequency in the postsynaptic cell to 1090.4±532.1% of baseline frequency from before menthol application. This was significantly increased from non-transfected neurons or neurons transfected with only Syn-YFP (N = 13), which produced a mPSC frequency in menthol of 104.1±6.3% of baseline frequency (Figure 7B and 7C; Fisher's exact test, p<0.005). We conclude that it possible to depolarize presynaptic terminals directly with menthol, which suggests that functional TRPM8 protein is expressed on or very near axon terminals.

Since synaptic transmission is depressed in TRPV1-transfected neurons (Figure 6), we tested whether TRPV1 is expressed near synaptic sites and whether presynaptic release is hindered. The baseline mPSC frequency, like with TRPM8 transfection, varied greatly in the postsynaptic targets of control (0.5–93.2 Hz) and, less so, TRPV1-transfected (1.0–35.1 Hz) neurons, although the mean baseline mPSC frequency (9.5±7.1 Hz and 15.3±5.2 Hz, respectively) did not differ significantly. When 500 nM capsaicin was applied to Syn-YFP-positive puncta of TRPV1-transfected neurons apposed to non-transfected neurons in the presence of 0.5 µM TTX, the mPSC frequency increased to 1016.8±323.2% of baseline frequency (N = 7; Figure 7D). This significantly differed from control neurons not in contact with transfected neurons (N = 9), for which capsaicin induced a mPSC frequency of only 121.3±34.7% of baseline frequency (Figure 7D; Fisher's exact test, p<0.01). Like with TRPM8-transfected neurons, this suggests that functional TRPV1 protein is expressed on or very near axon terminals.

## Discussion

In this study we compared transfection of cultured hippocampal neurons with TRPV1 or TRPM8 nonselective cation channels, which both allow specific activation of a small subset of neurons within the neuronal network (Figure 1, Figure 3). Although TRPM8 activation mediates similar current densities to TRPV1 (Figure 4), TRPV1 has larger secondary effects on cell function, including interference with endogenous synaptic transmission in the absence of exogenous agonist stimulation (Figure 6) and overt calcium-dependent toxicity with agonist exposure (Figure 5). Our results highlight substantial differences between two related channels and suggest that care is needed in investigating secondary effects of heterologous channels before employing them as tools.

Our observations suggest that transfection of TRPV1 alone, in the absence of capsaicin stimulation, has important effects on neuronal function. Although we did not detect overt cell loss or swelling of capsaicin-naïve TRPV1-transfected cells in estimates of transfection efficiency 24 hr following transfection, cell-attached patch recordings from TRPV1-transfected cells exhibited markedly more seal instability (Figure 1C) and spontaneous rupture compared with TRPM8-transfected cells (Figure 3C). In addition, evidence suggests that at least some TRPV1 channels are activated in the absence of exogenous capsaicin application (Figure 2). It is possible that even more neurons contain activated TRPV1 channels than we measured since culture medium contains many factors that are not included in defined recording saline. Seal instability could represent an early sign of toxicity from low-probability non-ligand-gated channel openings and/or from low-level presence of endogenous activators in the culture medium (e.g. pH, lipid compounds, or heat) in the absence of capsaicin [40]. Since the effects of TRP channel agonists are enhanced at more depolarized membrane potentials [41], it is possible that spontaneous activity may shift the heat sensitivity of TRPV1 enough to cause a persistent leak current while in the incubator. However, this cannot explain the persistent current measured electrophysiologically (Figure 2) since all recordings were performed at room temperature. Alternatively, TRPV1 overexpression itself (in the absence of channel activity) could promote these signs of compromised membrane integrity.

These same explanations (low-level channel openings or channel overexpression itself) may account for the strong synaptic depression observed in TRPV1-transfected neurons in the absence of capsaicin stimulation (Figure 6). Since these synaptic changes were measured in autaptic neurons, they could be presynaptic or postsynaptic. Because action potential-independent transmitter release from TRPV1-tranfected presynaptic terminals appeared intact (Figure 7), autaptic depression may represent a postsynaptic change, but it is also possible that TRPV1 expression disrupts presynaptic action potential propagation (e.g. as a result of a leaky axonal membrane) and/or action potential coupling to transmitter release. We have previously found that neuronal depolarization in cultured hippocampal neurons leads to a homeostatic decrease in EPSC amplitude and mEPSC frequency [42]. It is possible that low-level TRPV1 channel openings induce a similar phenomenon, although we might have expected stronger evidence of reduced mPSC frequency from TRPV1-transfected neurons (Figure 7). One possibility is that contributions from non-transfected presynaptic cells reduced the sensitivity of this experiment to detect synaptic depression. The autaptic experiments did not suffer this limitation. Regardless of the mechanism, this autaptic depression in the absence of added exogenous agonist is a poorly controlled, unintended consequence of TRPV1 overexpression and represents a major disadvantage of heterologous TRPV1 expression.

Prolonged capsaicin-induced TRPV1 activation led to increased overt signs of cell toxicity (Figure 5B). This again contrasted with TRPM8-transfected cells (Figure 5C) despite similar normalized depolarizing currents (Figure 4). By activating TRPV1 in the absence of extracellular calcium, we determined that the toxicity most likely results from calcium influx into the cell (Figure 5B). Since ionotropic glutamate receptor blockers were present during these experiments, toxicity cannot be attributed to calcium influx through NMDA receptors via indirect increases in network excitability. Thus, the high calcium permeability of the TRPV1 channel relative to TRPM8 [27], [28] is likely a strong contributor to toxicity. These results are akin to higher toxicity of calcium-permeable NMDA receptor activation relative to AMPA receptor activation [36], [43], [44], [45]. It is possible that activation-dependent pore dilation, which occurs in TRPV1 [46], [47] but not TRPM8 [48], also participates in the stronger toxicity induced by capsaicin treatment. Using saturating concentrations of agonists for these treatments may have exacerbated toxicity, so it is possible that lower concentrations could be titered to produce significant depolarizations without concurrent toxicity or other secondary effects.

Together, the experiments in this study suggest serious disadvantages of TRPV1 overexpression. To complicate matters, recent evidence also suggests that functional TRPV1 is expressed endogenously in the central nervous system (e.g. the hippocampus) in addition to its well-known expression in the peripheral nervous system [49], [50], [51]. Use of heterologous channels for selective stimulation obviously requires the demonstrated absence of contributions from endogenous channels. In our experiments, we never observed capsaicin-induced effects in non-transfected neurons (e.g. Figure 1D and 1F, Figure 2, Figure 7D, and Figure S1B), suggesting that endogenous receptor had a negligible contribution. This lack of detection of endogenous TRPV1 activation may be due to differences in expression in cultured neurons or to experimental manipulations that, for whatever reason, did not measure the effects of endogenous channel activation.

TRPM8 appears to be a much better candidate than TRPV1 for studies utilizing heterologous channel expression. TRPM8 activation produced robust currents (Figure 3, Figure 4) but induced less toxicity than TRPV1 (Figure 5C) and did not alter baseline synaptic transmission (Figure 6). Additionally, we have demonstrated that TRPM8, like TRPV1, can be used to manipulate local presynaptic function. Agonist application to transfected neurites in the absence of action potentials increased miniature PSC frequency in the postsynaptic cell (Figure 7). This suggests that agonist application stimulates calcium entry into presynaptic terminals through the TRP channel and/or through local voltage-gated calcium channels to cause neurotransmitter release. Using menthol as a TRPM8 agonist, however, has its caveats; menthol induced a small current mediated by GABA_A_ receptors (Figure 3G, Figure S2). This current, however, was easily reduced by applying the GABA_A_ antagonist picrotoxin, and other agonist choices for TRPM8 activation exist [28]. We conclude that TRPM8 is superior for most purposes and has fewer caveats than TRPV1.

Our study has shown how two related ligand-gated ion channels, TRPV1 and TRPM8, behave differently when expressed in the same neuronal preparation. Although we have shown that these channels, but especially TRPM8, are useful tools for controlling neuronal activity, this study also emphasizes the care needed in characterizing the nature of a newly introduced tool like heterologous channel expression. In the future, it will be important to ensure that other heterologous channels or proteins used to manipulate neuronal activity behave as predicted, especially after long periods of activation. Controlling neuronal activity in subsets of neurons within a network will facilitate studies of neuronal development, functional anatomy, synaptogenesis, and synaptic plasticity as well as provide a framework for studies manipulating neuronal populations *in vivo*.

## Materials and Methods

### Tissue culture and transfection

All experimental procedures involving animals were performed using protocols approved by the Washington University in St. Louis School of Medicine Animal Studies Committee as well as the *Guide for the Care and Use of Laboratory Animals* published by the U.S. National Institutes of Health. Primary hippocampal neuron cultures were prepared as previously described (Mennerick *et al*., 1995). Briefly, postnatal day 0–3 rat pups were anesthetized with isofluorane and decapitated. Hippocampi were removed, cut into 500 µM-thick transverse slices, and treated enzymatically with 1 mg/mL papain. Cells were then mechanically dissociated and plated as mass cultures (∼650 cells/mm^2^ onto a uniform layer of collagen) or as microcultures (∼100 cells/mm^2^ onto “islands” made of collagen droplets). The plating medium used was Eagle's medium (Invitrogen, Carlsbad, CA, USA) supplemented with heat-inactivated horse serum (5%), fetal bovine serum (5%), 17 mM D-glucose, 400 µM glutamine, 50 U/mL penicillin, and 50 µg/mL streptomycin. Cultures were housed in a humidified incubator at 37°C under controlled atmospheric conditions (5% CO_2_/95% air). To inhibit cell division 3–4 days after plating, 6.7 µM cytosine arabinoside was added. Half of the culture medium was replaced with Neurobasal medium (Invitrogen) with B27 supplement 4–5 days after plating. Transfections were performed 8–12 days after plating using a DNA∶Lipofectamine 2000 (Invitrogen) ratio of 1∶1.5–2 according to the manufacturer's protocol. Dishes were transfected with synaptophysin-YFP (0.5–2.5 µg), TRPV1 (2 µg), and/or TRPM8 (1.5 µg). Experiments were performed usually 1 day, but up to 3 days, after transfection.

### Electrophysiology

Whole-cell voltage-clamp and cell-attached patch experiments were performed using an Axopatch 200B amplifier (Molecular Devices, Sunnyvale, CA, USA), Digidata 1322A acquisition board (Molecular Devices), and pClamp software (Molecular Devices). All recordings were made in voltage-clamp mode at room temperature (∼25°C). Whenever solutions were acutely applied to neurons, a multi-barrel perfusion system was used with the perfusion port placed <0.5 mm from the neuron under study. For all experiments, controls consisted of either neurons from sibling cultures plated the same day or non-transfected neurons within the same dish as the transfected neurons under study unless otherwise described. All experiments, except for those depicted in Figure 6, were performed in conventional mass cultures.

Membrane potential was typically held at −70 mV for experiments, unless otherwise indicated. Electrode resistances of 6–11 MΩ were used for cell-attached patch recordings. For all other recordings, electrode resistances were 2.5–6 MΩ. A 5 kHz low-pass filter was used for all experiments except cell-attached patches in which 1 kHz filtering was utilized. Access resistance was compensated 85–100% for all experiments except where small currents were measured: cell-attached patches, miniature postsynaptic current recordings, acute agonist applications to non-transfected neurons, and acute ruthenium red applications to transfected and non-transfected neurons. Action potential stimulation for the autaptic recordings was achieved by 1.5 ms voltage pulses from −70 mV to 0 mV [39].

Extracellular recording media consisted of 138 mM NaCl, 4 mM KCl, 2 mM CaCl_2_, 1 mM MgCl_2_, 10 mM glucose, 10 mM HEPES, and 25 µM D-APV (Tocris, Bristol, UK) with a pH of 7.25. For all agonist-induced current recordings (i.e. excluding microisland autaptic current and miniature postsynaptic current recordings), 1 µM NBQX (Tocris) was added to the extracellular media to inhibit secondary glutamate-mediated synaptic currents. For most experiments, internal pipette solution consisted of 130 mM cesium methanesulfonate (CsMeSO_4_), 4 mM NaCl, 0.5 mM CaCl_2_, 5 mM EGTA, and 10 mM HEPES at a pH of 7.25. For cell-attached patch experiments, however, 140 mM K-gluconate was substituted for CsMeSO_4_, and for microisland autaptic recordings, 140 mM KCl was substituted for CsMeSO_4_ to provide similar driving force on excitatory and inhibitory postsynaptic currents (PSCs) at the holding potential of −70 mV.

For some experiments, agonist-induced responses were compared with responses elicited by 30 mM KCl application. Prolonged exposure (4 hr) to 30 mM KCl has been shown to induce strong synaptic change in cultured hippocampal neurons and clamps the membrane potential near −20 mV [32], [42]. Normalizing the agonist-induced current to the KCl current, therefore, controls for variability in absolute current size due to neuronal geometry and provides a within-cell comparator to a calibrated depolarizing stimulus.

### Epifluorescence imaging and cell counts

All imaging was performed using an Eclipse TE2000-S inverted microscope (Nikon, Melville, NY, USA) with epifluorescence provided by a metal halide lamp. Light was routed through Chroma filter set 41001 (Chroma Technology Corp., Rockingham, VT, USA), with an HQ480/40 nm excitation filter and an HQ535/50 nm emission filter, and through Chroma filter set 41002 (Chroma Technology Corp.), with an HQ535/50 nm excitation filter and an HQ610/75 nm emission filter. Images were acquired using Metamorph software (Universal Imaging, Downingtown, PA, USA) with a cooled 12-bit CCD camera (Photometrics, Tucson, AZ, USA) through a 40X objective (0.6 numerical aperture). To determine transfection efficiency, the number of transfected and non-transfected neurons was counted in 10 randomly-selected fields for each culture dish by an observer naïve to the DNA combination. For cell health experiments, dishes were fixed for 10 min in 4% paraformaldehyde/0.2% glutaraldehyde after agonist treatments. After washing 3 times in phosphate-buffered saline, transfected neurons were assessed for health under 40× magnification by an observer naïve to both the treatment conditions and the DNA combination.

### Data analysis

For all electrophysiological experiments, data were analyzed using Clampfit 9.2 (Molecular Devices) and Excel (Microsoft, Redmond, WA, USA) software. Graphs and traces were plotted using SigmaPlot software (SPSS Science, Chicago, IL, USA). Transfection efficiency was calculated by averaging the percentage of transfected neurons in each dish. This percentage was determined by dividing the number of transfected neurons by the total number of neurons counted in 10 fields and multiplying by 100. For Figure 1 and Figure 3, an action potential was counted if there was a 1–10 pA transient and primarily negative current deflection above noise lasting ≥1 ms. Except for electrically-evoked currents elicited in autaptic neurons in microisland culture, current amplitudes were measured by averaging ∼500 ms of baseline and subtracting this from the average of ∼500 ms around the peak and/or steady state amplitude. For Figure 4, the current ratio is measured by dividing the agonist-induced current amplitude by the KCl-induced current amplitude. For Figure 6, the baseline leak amplitude was subtracted from the peak amplitude for at least 3 traces from each cell and averaged. For autaptic experiments, cells with leak currents >300 pA were excluded from analysis. To determine the percentage of healthy transfected neurons after agonist treatment, the number of healthy neurons was divided by the total number of transfected neurons counted in a particular culture dish and multiplied by 100. Miniature postsynaptic current frequency was calculated from 10 s of recording using MiniAnalysis software (Synaptosoft, Decatur, GA, USA). Baseline frequency was measured before application of agonist while the frequency in agonist was measured beginning ∼1 s after application onset. The percentage of baseline frequency was calculated by dividing the frequency in agonist by the frequency at baseline and multiplying by 100. Data are presented in the text and figures as mean±SEM unless otherwise stated. Student's unpaired *t* test, ANOVA, post hoc Tukey honestly significant difference test for multiple comparisons, Tukey-Kramer method for unequal sample sizes, binomial test, and Fisher's exact test were used to determine statistical significance where described in the text. A p-value of less than 0.05 was required for significance.

### Materials

Alexa Fluor 568 was purchased from Invitrogen. Synaptophysin-YFP and TRPV1 constructs were graciously provided by Dr. Ann Marie Craig (University of British Columbia) and Dr. David Julius (University of California San Francisco), respectively. TRPM8 has been used previously [52], [53]. The calcium-free culture medium used for toxicity experiments was made by the Tissue Culture Support Center at Washington University by excluding 1.8 mM calcium chloride from Neurobasal medium recipe [54]. Unless otherwise stated, all other chemicals and reagents were purchased from Sigma (St. Louis, MO, USA). When DMSO concentration in experimental solutions was >0.1% after addition of reagents from DMSO stock solutions, control and experimental conditions were matched for final DMSO concentration.

## Supporting Information

Figure S1 Agonist application to transfected neurons causes action potentials and strong depolarization. (A) Example of a TRPV1-transfected neuron exposed to 5 s acute 30 mM KCl (black trace) or 500 nM capsaicin (red trace). Neurons were recorded in whole-cell current-clamp as described in Text S1 after adjusting the baseline membrane potential to −65 mV with small bias current when necessary. Although capsaicin-induced voltage changes were slow to return to baseline, they did so within ∼20 s. (B) The same as A except recording from a non-transfected neuron in the same culture. (C) Example of a TRPM8-transfected neuron exposed to 5 s acute 30 mM KCl (black trace) or 100 µM menthol (red trace). Neurons were recorded in current clamp after adjusting the baseline membrane potential to −65 mV as described in (A). (D) The same as (C) except recording from a non-transfected neuron in the same culture.
(0.24 MB TIF)

Figure S2 Menthol application to non-transfected neurons induces a current that changes direction with the chloride gradient. (A) Example of a non-transfected neuron recorded in whole-cell voltage-clamp as described in Text S1 in the presence of 0.5 µM tetrodotoxin (TTX) with a cesium methanesulfonate internal pipette solution held at various membrane potentials during a local 5 s 100 µM menthol application. Note the change in current direction with potentials above and below ∼−60 mV. (B) Example of a non-transfected neuron recorded in the presence of 0.5 µM TTX with a cesium chloride internal pipette solution held at various membrane potentials during a local 5 s 100 µM menthol application. Note the change in current direction with potentials above and below ∼−20 mV. These data are consistent with menthol gating a small GABAA receptor-mediated current in non-transfected hippocampal neurons (see Figure 3G).
(0.30 MB TIF)

Text S1 Supporting methods.
(0.03 MB DOC)
