# Supplementary figures and images for: Comparative Effects of Heterologous TRPV1 and TRPM8 Expression in Rat Hippocampal Neurons
Source: PLoS One. 2009 Dec 4;4(12):e8166. doi: 10.1371/journal.pone.0008166 (PMC2780724; doi:10.1371/journal.pone.0008166)

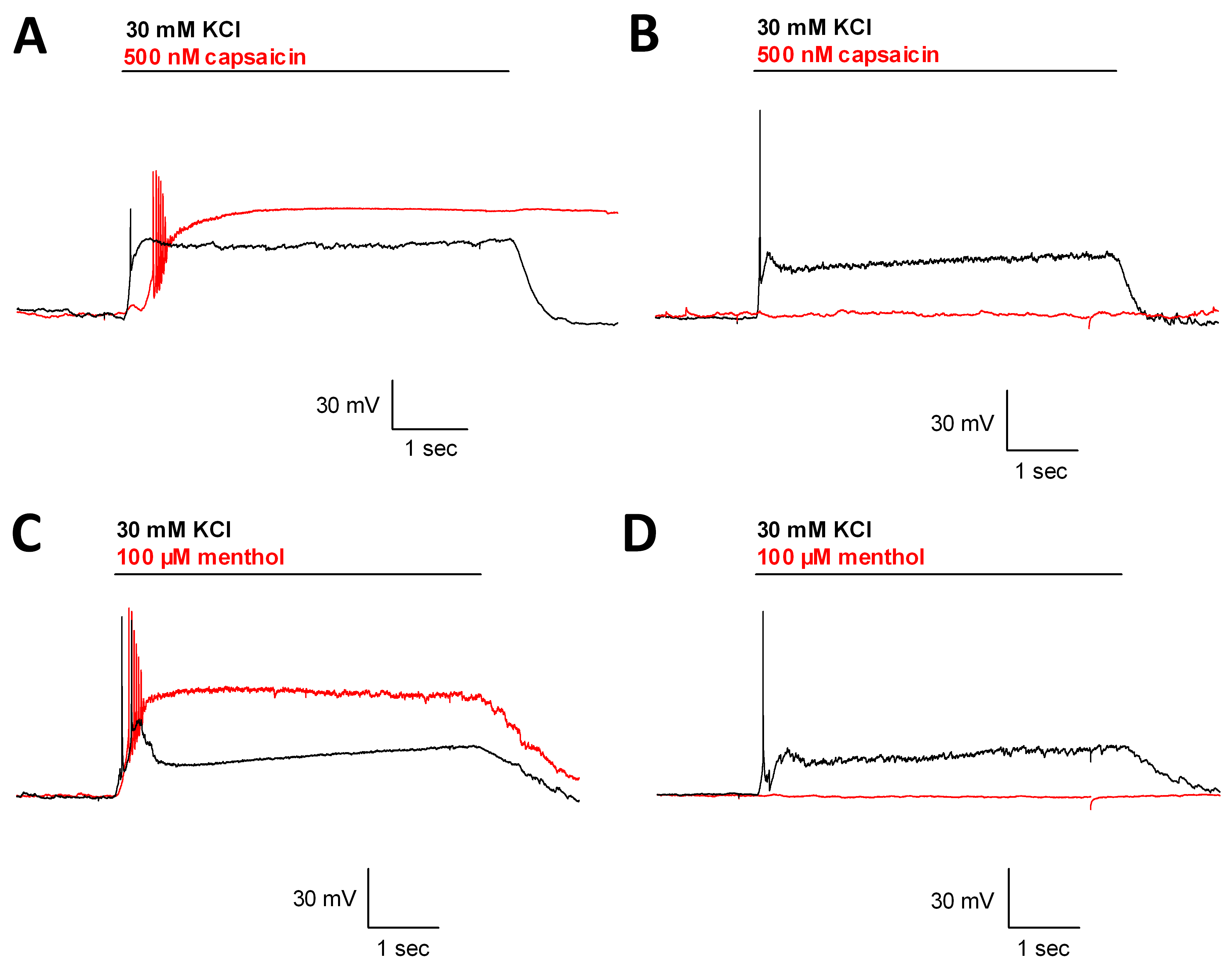

Supplement: Figure S1 — Agonist application to transfected neurons causes action potentials and strong depolarization. (A) Example of a TRPV1-transfected neuron exposed to 5 s acute 30 mM KCl (black trace) or 500 nM capsaicin (red trace). Neurons were recorded in whole-cell current-clamp as described in Text S1 after adjusting the baseline membrane potential to −65 mV with small bias current when necessary. Although capsaicin-induced voltage changes were slow to return to baseline, they did so within ∼20 s. (B) The same as A except recording from a non-transfected neuron in the same culture. (C) Example of a TRPM8-transfected neuron exposed to 5 s acute 30 mM KCl (black trace) or 100 µM menthol (red trace). Neurons were recorded in current clamp after adjusting the baseline membrane potential to −65 mV as described in (A). (D) The same as (C) except recording from a non-transfected neuron in the same culture. (0.24 MB TIF) [file pone.0008166.s001.tif]

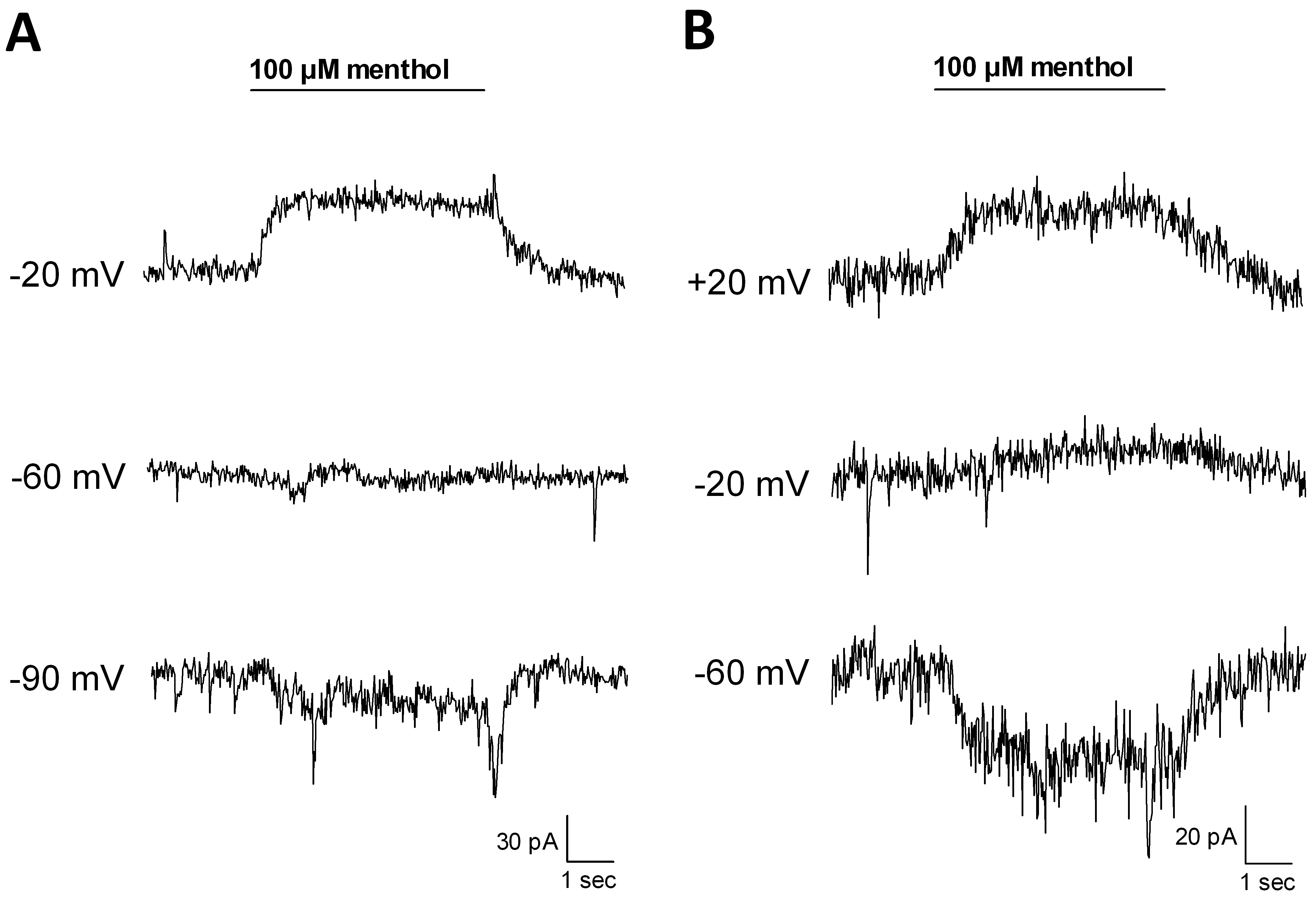

Supplement: Figure S2 — Menthol application to non-transfected neurons induces a current that changes direction with the chloride gradient. (A) Example of a non-transfected neuron recorded in whole-cell voltage-clamp as described in Text S1 in the presence of 0.5 µM tetrodotoxin (TTX) with a cesium methanesulfonate internal pipette solution held at various membrane potentials during a local 5 s 100 µM menthol application. Note the change in current direction with potentials above and below ∼−60 mV. (B) Example of a non-transfected neuron recorded in the presence of 0.5 µM TTX with a cesium chloride internal pipette solution held at various membrane potentials during a local 5 s 100 µM menthol application. Note the change in current direction with potentials above and below ∼−20 mV. These data are consistent with menthol gating a small GABAA receptor-mediated current in non-transfected hippocampal neurons (see Figure 3G). (0.30 MB TIF) [file pone.0008166.s002.tif]
