# Supplementary material for: Comparative Effects of Heterologous TRPV1 and TRPM8 Expression in Rat Hippocampal Neurons
Source: PLoS One. 2009 Dec 4;4(12):e8166. doi: 10.1371/journal.pone.0008166 (PMC2780724; doi:10.1371/journal.pone.0008166)
Supplement: Text S1 — Supporting methods. (0.03 MB DOC) [file pone.0008166.s003.doc]

**Text S1**

**Materials and Methods**

Electrophysiology:

Whole-cell current-clamp experiments were performed on DIV 12-13 neurons in mass culture 1 day after transfection with TRPV1 or TRPM8. External recording media contained 138 mM NaCl, 4 mM KCl, 2 mM CaCl_2_, 1 mM MgCl_2_, 10 mM glucose, 10 mM HEPES, 25 µM D-APV, and 1 μM NBQX (pH = 7.25). Internal pipette solution contained 140 mM K-gluconate, 4 mM NaCl, 0.5 mM CaCl_2_, 5 mM EGTA, and 10 mM HEPES at a pH of 7.25. Recording electrode resistances of 6-9 MΩ and a 10 kHz low-pass filter were used for these experiments. The membrane potential of each cell was manually adjusted to -65 mV by injecting bias current before the start of recording.

For voltage-clamp experiments measuring small menthol-induced currents in non-transfected neurons, methods from the main text were utilized except that an internal pipette solution of 130 mM cesium chloride, 3 mM NaCl, 0.5 mM CaCl_2_, 5 mM EGTA, and 10 mM HEPES (pH of 7.25) was used as described in addition to the solution with 130 mM cesium methanesulfonate, 4 mM NaCl, 0.5 mM CaCl_2_, 5 mM EGTA, and 10 mM HEPES (pH of 7.25). Also, the external recording media was as described above except that 0.5 μM tetrodotoxin was added. The multi-barrel perfusion system described in the main text was utilized for locally applying compounds.
